# Association mapping of stem rust race TTKSK resistance in US barley breeding germplasm

**DOI:** 10.1007/s00122-014-2297-8

**Published:** 2014-04-08

**Authors:** H. Zhou, B. J. Steffenson, Gary Muehlbauer, Ruth Wanyera, Peter Njau, Sylvester Ndeda

**Affiliations:** 1 Department of Plant Pathology, University of Minnesota, St. Paul, MN USA; 2 Department of Agronomy and Plant Genetics, University of Minnesota, St. Paul, MN USA; 3 Department of Plant Biology, University of Minnesota, St. Paul, MN USA; 4 Kenya Agricultural Research Institute, Njoro, Kenya; 5 East African Maltings Limited, Molo, Kenya

## Abstract

*****Key message***:**

**Loci conferring resistance to the highly virulent African stem rust race TTKSK were identified in advanced barley breeding germplasm and positioned to chromosomes 5H and 7H using an association mapping approach.**

**Abstract:**

African races of the stem rust pathogen (*Puccinia graminis* f. sp. *tritici*) are a serious threat to barley production worldwide because of their wide virulence. To discover and characterize resistance to African stem rust race TTKSK in US barley breeding germplasm, over 3,000 lines/cultivars were assessed for resistance at the seedling stage in the greenhouse and also the adult plant stage in the field in Kenya. Only 12 (0.3 %) and 64 (2.1 %) lines exhibited a resistance level comparable to the resistant control at the seedling and adult plant stage, respectively. To map quantitative trait loci (QTL) for resistance to race TTKSK, an association mapping approach was conducted, utilizing 3,072 single nucleotide polymorphism (SNP) markers. At the seedling stage, two neighboring SNP markers (0.8 cM apart) on chromosome 7H (11_21491 and 12_30528) were found significantly associated with resistance. The most significant one found was 12_30528; thus, the resistance QTL was named *Rpg*-*qtl*-*7H*-*12_30528.* At the adult plant stage, two SNP markers on chromosome 5H (11_11355 and 12_31427) were found significantly associated with resistance. This resistance QTL was named *Rpg*-*qtl*-*5H*-*11_11355* for the most significant marker identified. Adult plant resistance is of paramount importance for stem rust. The marker associated with *Rpg*-*qtl*-*5H*-*11_11355* for adult plant resistance explained only a small portion of the phenotypic variation (0.02); however, this QTL reduced disease severity up to 55.0 % under low disease pressure and up to 21.1 % under heavy disease pressure. SNP marker 11_11355 will be valuable for marker-assisted selection of adult plant stem rust resistance in barley breeding.

**Electronic supplementary material:**

The online version of this article (doi:10.1007/s00122-014-2297-8) contains supplementary material, which is available to authorized users.

## Introduction

Stem rust is one of the most serious diseases of small grain cereals because it is capable of completely destroying crops across a large area in a short period of time. Although wheat has historically been most affected by stem rust, barley also can suffer damage due to this disease. In barley, significant yield losses have been reported in the major production areas of the northern Great Plains in the United States and Canada (Harder and Dunsmore 1991) and also in southern Queensland and northern New South Wales in Australia (Dill-Macky et al. 1991). Barley is host to two different stem rust pathogens: *Puccinia graminis* Pers.:Pers. f. sp. *tritici* Eriks. and E. Henn., the wheat stem rust pathogen, and *P. graminis* Pers.:Pers. f. sp. *secalis* Eriks. and E. Henn., the rye stem rust pathogen, with the former being most important (Steffenson 1992). Since the mid-1940s, losses due to stem rust have been minimal in barley cultivars grown in North America due to the deployment of the resistance gene *Rpg1* (Steffenson 1992).

In 1999, a new race of *P. graminis* f. sp*. tritici* named TTKSK (former designation: TTKS with isolate synonym of Ug99) was described from Uganda and alarmed scientists because it was virulent on *Sr31*, a widely used stem rust resistance gene in wheat (Pretorius et al. 2000). More than 80 % of the world’s wheats are susceptible to this race (Singh et al. 2006). From its initial discovery in Uganda, race TTKSK has spread to other countries in Africa and has gained a foothold in the Middle East (Iran) (Nazari et al. 2009; http://rusttracker.cimmyt.org/?page_id=22). A recent study also revealed that race TTKSK is capable of infecting more than 95 % of world’s barleys (Steffenson et al. 2013), including those carrying the *Rpg1* resistance. Because of its wide virulence on both wheat and barley and its spread, race TTKSK is a serious threat to global cereal production. Fungicide applications can reduce the impact of stem rust; however, the expense and possible environmental concerns of such treatments have led to a focus on host resistance.

In barley, five genes for resistance to the wheat stem rust pathogen have been reported. *Rpg1*, derived from Peatland (CIho 5267), Chevron (CIho 1111), or Kindred (CIho 6969) (Powers and Hines 1933; Shands 1939; Steffenson 1992) is present in every commercial malting cultivar in the Upper Midwest region of the United States and has provided broadly effective resistance for over 60 years (Steffenson 1992). *Rpg2* was identified in the accession Hietpas 5 (CIho 7124) (Patterson et al. 1957) and *Rpg3* from the Ethiopian landrace PI382313 (Jedel et al. 1989; Jedel 1990). The recessive gene *rpg4* was identified in line Q21861 (PI 584766) (Jin et al. 1994). Finally, *rpg6*, another recessive gene, was identified in 212Y1, a barley line containing an introgression from *Hordeum* *bulbosum* (Fetch et al. 2009).

Three genes have been identified for rye stem rust resistance in barley. The one identified in barley accession skinless is dominant in gene action (Luig 1957). A recessive gene, given the provisional designation of *rpgBH*, was identified in Black Hulless (CIho 666) (Steffenson et al. 1984). Finally, a third gene *Rpg5* (formerly designated as *RpgQ*) was identified in Q21861 and is closely linked to *rpg4* on the long arm of chromosome 5H (Brueggeman et al. 2008; Sun et al. 1996; Sun and Steffenson 2005).

Among all these reported stem rust resistance genes, only *Rpg1* has been deployed in agriculture, although genes at the *rpg4*/*Rpg5* complex and *Rpg3* have been used in some breeding programs. The *rpg4/Rpg5* complex comprises the only major genes known to confer resistance to race TTKSK in barley (Steffenson et al. 2009). Several quantitative trait loci (QTL) conferring seedling and adult plant resistance to race TTKSK were recently reported in line Q21861 (Moscou et al. 2011). However, only the QTL at the *rpg4/Rpg5* locus was consistently detected in all environments.

Several gene deployment strategies have been used to control rust diseases in small grain cereals over the past 80 years. The first one utilized single major resistance genes, but control was often short-lived due to the emergence of new virulent races in the stem rust population (Johnson 1984; Steffenson 1992). A notable exception has been the *Rpg1* gene, which has protected barley from major losses for over 60 years. The repeated “boom and bust” cycles brought on by single gene deployment in wheat led to the alternative strategy of combining or “pyramiding” multiple major resistance genes into cultivars (Dangl and Jones 2001; Pink 2002). This strategy has been highly effective in controlling stem rust since the 1950s in the northern Great Plains of North America and Australia (Leonard and Szabo 2005; Line and Chen 1995; Park 2007). Another gene deployment strategy involves the use of partial or incomplete adult plant resistance, which is often controlled by several minor effect genes. Partial resistance allows the pathogen to infect the plant and ramify to some extent. However, the selection pressure exerted on the pathogen to overcome this resistance is minimized, thereby extending the life of the resistance (Johnson 1984). One such example is the durable leaf rust resistance in spring wheats grown in North America, Mexico and Australia (Kolmer 1996) where a high level of resistance was achieved by accumulating 4–5 genes (Singh et al. 2005).

Given the serious threat that race TTKSK and its variants pose to wheat and barley production worldwide, research is needed to identify and genetically characterize new sources of resistance. Additionally, more efficient tools (e.g., marker-assisted selection or MAS) must be developed and utilized to hasten the development of resistant cultivars with superior yield, quality, and agronomic traits. There are two major quantitative genetic approaches that can be used to identify loci associated with traits such as stem rust resistance. One is linkage mapping or biparental mapping (Lander and Botstein 1989), which uses the co-segregation of marker alleles with phenotypic data within progenies to identify QTL that contain causal variants. In linkage or biparental mapping populations, the progeny are usually just a few generations advanced from the first cross between two parents, which results in a broad extent of linkage disequilibrium (LD). Thus, distant markers are usually found to co-segregate with the causal variant. Although linkage mapping has proven successful in identifying QTL for hundreds of traits in many plant species (Doerge 2002; Mackay et al. 2009; Mauricio 2001), the identified QTL region can extend over several or more centiMorgans (cM) and contain hundreds of candidate genes. Therefore, QTL introgressed via MAS may suffer from linkage drag, the hitch-hiking of deleterious genes linked to target genes under selection. In addition, the construction of mapping populations through controlled crosses is time-consuming, which further restricts the use of linkage mapping. An alternative method for identifying QTL is association mapping (AM) or LD mapping. AM seeks to identify specific causal variants linked to phenotypic polymorphisms in more diverse panels of germplasm. AM panels are usually many generations removed from a common ancestor. Recombination events occurring throughout the evolutionary history of the panel contribute to the breakage of LD blocks within the genome. Thus, LD decays much faster in AM panels than linkage mapping populations (Rafalski 2002). AM can therefore achieve a higher resolution of causative trait polymorphism than linkage mapping, thereby reducing linkage drag. This mapping approach has been successfully used in various plant species to identify markers associated with many different traits, including disease resistance (Breseghello and Sorrells 2006; Cockram et al. 2010; Cuesta-Marcos et al. 2010; Kraakman et al. 2004, 2006; Roy et al. 2010; Skøt et al. 2007; Thornsberry et al. 2001; Yu et al. 2011).

The threat that race TTKSK poses to US barley production can be best mitigated by developing resistant cultivars. Currently, little is known regarding the reaction of US barley breeding germplasm to this widely virulent race. If resistance were discovered and genetically characterized in such advanced lines, the time needed to produce a resistant cultivar acceptable by industry would be greatly reduced. The Barley Coordinated Agricultural Project (BCAP) (http://www.barleycap.org) was established in 2006 to apply the tools of genomics to plant breeding. It comprises 3,840 advanced US breeding lines and also cultivars that have been genotyped with over 3,000 single nucleotide polymorphism (SNP) markers. Thus, the BCAP provides a unique opportunity to identify and genetically characterize loci contributing to this new biotic threat through AM. Thus, the objectives of this research were to: (1) characterize the reactions of US barley breeding lines to race TTKSK at the seedling and adult plant stages and (2) map the loci contributing to TTKSK resistance.

## Materials and methods

### Plant materials

The mapping panel used in this AM study was developed by BCAP and consists of advanced breeding lines and also cultivars from ten US barley improvement programs: eight spring type and two winter/facultative type programs (Supplementary Table 1). Aside from yield, quality, and agronomic traits, these breeding programs focus on different end uses for barley such as malting, feed, and food. All lines were inbred to at least the F_4_ generation and were selected to be representative of each program. 96 lines were submitted from each of the ten breeding programs in each year of the project from 2006 to 2009. Thus, the total number of lines evaluated per year was 960 for a project total of 3,840. Since winter/facultative barleys could not be reliably grown to the heading stage in the field, adult plant phenotyping was only conducted on spring types (768 lines per year and 3,072 lines in total).

AM analyses for seedling resistance were conducted separately by year for the four individual panels of CAPI (2006 panel with 960 lines), CAPII (2007, 960 lines), CAPIII (2008, 960 lines), and CAPIV (2009, 960 lines) as well as the complete panel designated hereafter as CAP (3,840 lines). AM analyses for adult plant resistance were conducted separately by year for spring (S) barleys in the individual panels of CAPI-S (768 lines), CAPII-S (768 lines), CAPIII-S (768 lines), and CAPIV-S (768 lines) as well as the complete spring panel hereafter designated as CAP-S (3,072 lines).

### SNP genotyping

Lyophilized leaf tissue from a single plant selection of each barley line was sent to the USDA-ARS Biosciences Research Laboratory in Fargo, ND, for DNA extraction and genotyping. DNA was isolated according to standard procedures (Pallotta et al. 2003). Following the protocols of Illumina’s GoldenGate BeadArray Technology (Illumina, San Diego, CA, USA) (Fan et al. 2003), two barley oligonucleotide pool assays (BOPA1 and BOPA2) (Close et al. 2009) containing allele-specific oligos for a set of 3,072 SNPs were used to genotype the barley lines. All lines were genotyped by Illumina SNP technology (Gunderson et al. 2004) on the Illumina^®^BeadStation 500G.

### Phenotyping at the seedling stage

Seedling evaluations of CAPI and CAPII were conducted in the USDA-ARS Cereal Disease Laboratory at St. Paul during the winter months of 2006 and 2007, while CAPIII and CAPIV were evaluated in the Biosafety Level-3 Containment Facility on the St. Paul campus of the University of Minnesota during the same period in 2008 and 2009. Five seeds from each line were sown in plastic pots (7.6 × 7.6 × 10.8 cm, l × w × h) filled with a 50:50 mixture of native soil and potting mix (Sunshine MVP; Green Island Distributors, Inc., Riverhead, NY) and grown at 20–22 °C with a 14-h photoperiod (230–270 μmol photon/m^2^/s^1^ provided by 1,000 W sodium vapor lamps). Two susceptible controls, Hiproly (PI 60693) and Steptoe (CIho 15229), and one resistant control, Q21861, also were included multiple times in the experiment. Controlled release (Osmocote 14-14-14; Scott’s Company, Marysville, OH; 1.4 g per pot) and water-soluble fertilizer formulations (Peters Dark Weather 15-0-15; Scott’s Company; 0.1 g per pot) were applied at planting. Inoculations were made when the first leaves of plants were fully expanded, about 9 days after planting. Wheat stem rust race TTKSK (isolate 04KEN156/04) was used in all experiments. For inoculum increase, urediniospores were inoculated onto the susceptible wheat host McNair 701 (CI 15288) and after sporulation were collected, desiccated, and stored at −80 °C until needed. On the day of inoculation, urediniospores were removed from the freezer, heat shocked at 45 °C for 10 min, and then allowed to re-hydrate in an 80 % RH chamber for 1 h. Urediniospores were suspended in Soltrol oil (Phillips Petroleum, Bartlesville, OK; 14 mg/0.7 ml) and sprayed onto plants (0.14 mg/plant) using a rust inoculator pressurized by an air pump (27.5 kPa). A small electric fan was then used to hasten the evaporation of the oil carrier from leaf surfaces to reduce phytoxicity. After inoculation, plants were moved into mist chambers where ultrasonic humidifiers were run for 30 min to establish a thin layer of free moisture on the leaf surfaces. Thereafter, the humidifiers were set to come on for 2 min every hour to maintain leaf wetness. During this time, plants were kept at 18–22 °C and near 100 % RH in the dark. After 16–18 h, lights (150–250 μmol photon/m^2^/s provided by 400 W high pressure sodium vapor lamps) were turned on, and the humidifiers were set to come on for 15 min every hour. Additionally, the chamber doors were opened partway to prevent excessive heat buildup. After 2 h, the misters were turned off, and the chamber doors were fully opened to facilitate the slow drying of moisture from the plant surfaces. Plants were then transferred to the greenhouse under the conditions previously described. The experiment was conducted using a completely randomized design and included two replicates.

At 14–17 days post-inoculation, stem rust infection types (ITs) were assessed on the first leaves of plants using a 0–4 rating scale. The IT scale used for barley is a modification of the one developed for wheat by Stakman et al. (1962) and is based primarily on uredinial size as described by Miller and Lambert (1955). Two or more ITs were frequently observed on individual plants of lines (i.e., a mesothetic reaction) challenged by *P. graminis* f. sp. *tritici*; thus, all of the ITs observed on lines were recorded in order of their prevalence. For AM analysis, these categorical phenotype data were transformed to numeric data as follows: IT “0” was coded as 0.0; IT “0;” or “;” as 0.5, IT “1” as 2.0, IT “2” as 3.0, IT “3^−^”as 3.5, IT “3” as 4.0, and IT “3^+^” as 4.5. IT “4” was not observed, but would be coded as 5.0. The numeric values assigned to the ITs reflect biologically and epidemiologically important differences in the host–parasite interaction. For example, the larger 1.5 unit difference assigned between IT 0; or ; and IT 1 reflects the biologically significant host reaction of a hypersensitive fleck with no sporulation vs. a host reaction with clear sporulation. Similarly, the 1.0 unit difference assigned between IT 1 and IT 2 reflects the smaller uredinial size and presence of distinct necrosis in the former vs. a larger uredinial size and the presence of distinct chlorosis in the latter. Thereafter, smaller 0.5 unit differences were assigned to ITs from 2 to 3^+^ because these reactions just reflect minor differences in uredinial size. The final numeric disease score for the line was then calculated from these transformed ITs after a multiplier was applied based on the general proportion of ITs as calculated from many infected leaf samples (Table 1). Briefly, if only one IT was observed on a line, the transformed numeric value of that IT was multiplied by 1.0 and the resulting value was used as the final numeric disease score. If two or three ITs were observed, the same transformation function was used for the IT scores, except that the respective multipliers of 0.75 + 0.25 and 0.60 + 0.30 + 0.10 were applied and summed to achieve the final numeric disease scores. Values of the multipliers for the two and three IT cases were based on observations of many infected leaf samples. The mean numeric score of the two replicates of each line was used in the AM analysis.

**Table 1:** Formulae used in transforming seedling categorical IT data into numeric data for AM of race TTKSK resistance in US barley breeding germplasm

Multiplier for respective ITs			Formulae for numeric score
Most prevalent IT	Second most prevalent IT	Next most prevalent IT	
A^a^ × 100 %^b^	–	–	A
A × 75 %	B × 25 %	–	0.75A + 0.25B
A × 60 %	B × 30 %	C × 10 %	0.6A + 0.3B + 0.1C

^a^A, B or C represent numeric values from 0.0 to 4.5 for the most prevalent IT, second most prevalent IT and next most prevalent IT, respectively, which were assigned to categorical ITs. Categorical IT “0” was coded as 0.0; IT “0;” or “;” as 0.5, IT “1” as 2.0, IT “2” as 3, IT “3^−^”as 3.5, IT “3” as 4.0, and IT “3^+^” as 4.5

^b^Barley commonly exhibits mesothetic reactions, i.e., a mixture of different IT on the same leaf. The multiplier after A, B, and C were weighted, reflecting the general proportions of the most prevalent IT, second most prevalent IT, and next most prevalent IT

### Phenotyping at the adult plant stage

Adult plant evaluations of each spring panel were conducted at the Kenya Agricultural Research Institute (KARI) in Njoro, Kenya. Lines were sown in June and assessed for rust reaction in October in both 2009 (CAPIII-S and CAPIV-S) and 2010 (CAPI-S and CAPII-S). For these tests, 20–25 seeds of each line were sown in short rowed plots (0.3 m long) in a completely randomized design with one replicate. The susceptible (Steptoe) and resistant (Q21861) controls were included in all experiments. The susceptible wheat variety “Red Bobs” (CItr 6255) was planted as a rust spreader row perpendicular to the line rows. To initiate rust infection in the nurseries, urediniospores suspended in water were injected by a syringe into the stems of wheat plants (1–3 plants/m) of the spreader row just prior to the boot stage of development. The percentage of stem and leaf sheath tissue infected by stem rust was estimated using the modified Cobb scale (0–100 %) (Peterson et al. 1948) at the mid-dough and hard-dough stages of development.

### Population structure, LD and AM analyses

Population structure and LD analyses in the different germplasm subsets used in this study were previously reported (Zhou et al. 2012).

A mixed linear model (MLM) implemented in software TASSEL (version 3.0) was used to detect associations between SNP markers and stem rust resistance in US barley breeding germplasm, both at the seedling and adult plant stages. The formula used was *y* = *Xβ* + *Pv* + *u* + *e*, where *y* is the vector of phenotypic values, *X* is the vector of SNP marker genotypes, *P* is the matrix of principle component vectors accounting for population structure and other covariate vectors, such as “year”, *β* is the coefficient of the marker effect being estimated, *v* is the coefficient of population structure, *u* is the vector of random effects, and *e* is the vector of residuals. In addition, the variances of *u* and *e* are given by: Var(*u*) = 2*KV*_g_ and Var(*e*) = *V*_R_, where *K* is the kinship matrix inferred from genotypes based on the proportion of shared allele values, *V*_g_ is the genetic variance, and *V*_R_ is the residual variance. Each SNP marker was then fit individually into the MLM, and a *p* value was generated. Concurrently, a naive model, where *y* = *Xβ*, was used to demonstrate the effectiveness of controlling structure with the MLM. The Benjamini-Hochberg (1995) false discovery rate (BH-FDR) of *q* value = 0.05 was used to correct for multiple comparisons using program *Q*VALUE (Storey 2002). AM was first performed on the individual yearly panels (CAPI to CAPIV and CAPI-S to CAPIV-S) and then in the complete panels (CAP and CAP-S) containing all possible data to identify resistance loci conferring seedling and adult plant resistance.

## Results

### Stem rust phenotype data

**Fig. 1:**
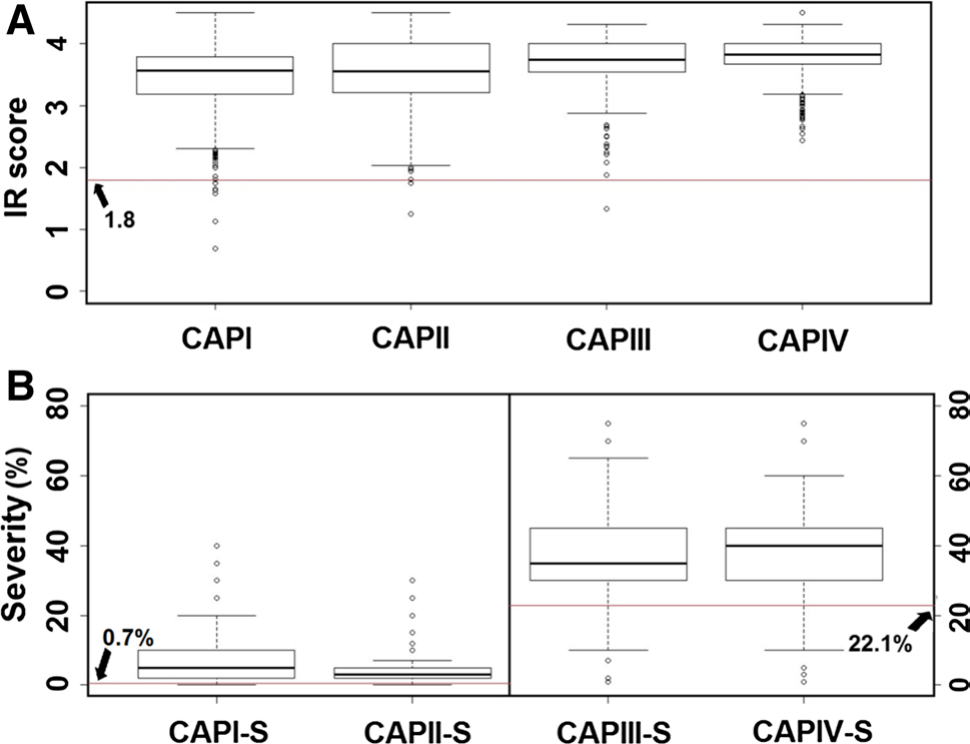
Distribution of stem rust race TTKSK seedling and adult plant phenotypic data of US barley breeding germplasm. CAPI, CAPII, CAPIII, and CAPIV are four individual panels from 2006 to 2009 comprising both spring and winter/facultative breeding lines and CAPI-S, CAPII-S, CAPIII-S, and CAPIV-S are four individual panels from the same years comprising only spring breeding lines. CAPIII-S and CAPIV-S were evaluated in a high disease year (2009), while CAPI-S and CAPII-S were evaluated in a low disease year (2010). Five statistics (*bars*) are represented in each boxplot from *bottom to top*: the smallest observation, lower quartile, median, upper quartile, and largest observation, respectively. Data points positioned outside this range are extreme values. **a** Boxplots of numeric seedling disease scores by individual yearly panels. The *horizontal line* indicates the mean plus one standard deviation of resistant control Q21861 **b** Boxplots of adult plant disease severity (0–100 %) by individual yearly spring panels. The *horizontal lines* indicate the mean plus one standard deviation of resistant control Q21861 in 2009 and 2010

In the seedling tests, the susceptible controls of Hiproly and Steptoe were included multiple times in all experiments to monitor the infection level and virulence phenotype of race TTKSK. In addition, line Q21861 was included as the resistant control because it reliably exhibits low ITs to race TTKSK under moderate temperatures. Moderate to high infection levels were observed on all BCAP germplasm and controls in each experiment, allowing for the reliable scoring of ITs. Mostly high ITs (IT mode of 3 with range of 3^−^2 to 3^+^; i.e., numeric score mode of 4 with range of 3.4–4.5) were observed on the susceptible controls, whereas only low ITs (IT mode of 0;1 with range of 0;–12; i.e., numeric score mode of 0.9 with range of 0.5–2.3) were observed on the resistant control in all experiments. For BCAP germplasm, the mean numeric disease scores were very similar across all four individual yearly panels and ranged from 3.4 (median = 3.6) in CAPI to 3.7 (median = 3.8) in CAPIV (Fig. 1a). The inter-quartile range (IQR) (distance between upper quartile and lower quartile values, a measure of variability in the data) was low across panels and ranged from 0.3 in CAPIV to 0.8 in CAPII (Fig. 1a).

Only 12 (0.3 %) lines (1 each from AB, BA, N6, WA and UT, 3 from N2, and 4 from OR) exhibited a high level of seedling resistance, defined here as a numeric disease score equal to or less than 1.8, which is the mean plus one standard deviation of the disease score exhibited by resistant control Q21861 (Fig. 1a).

In the adult plant tests, the susceptible (Steptoe) and resistant (Q21861) controls were included in all experiments to monitor the infection level and virulence of stem rust race TTKSK. In general, infection levels were high in 2009 and relatively low in 2010, due to the variable weather conditions occurring at the nursery site. The mean stem rust severity on Steptoe was 45.2 % (range of 40–60 %) in 2009 and 22.4 % (range of 15–30 %) in 2010. Mean rust severity on the resistant control of Q21861 was 13.7 % (range of 2–20 %) in 2009 and 0.2 % (range of 0–1 %) in 2010. For BCAP germplasm, disease levels varied markedly between the 2 years of study. In 2009, the weather conditions were more favorable for rust development and mean severities of 37.4 % (median = 35.0 %) and 37.0 % (median = 30.0 %) were observed on CAPIII-S and CAPIV-S, respectively (Fig. 1b). In contrast, mean disease severities of 6.3 % (median = 5.0 %) and 4.7 % (median = 3.0 %) were observed for CAPI-S and CAPII-S, respectively, in 2010—a year that was unfavorable for rust development (Fig. 1b). A wider range of variation for rust severity was observed in the panels exposed to higher disease pressure as the IQR was 15.0 % for both CAPIII-S and CAPIV-S, but only 3.0 and 8.0 % for CAPI-S and CAPII-S, respectively (Fig. 1b).

In 2009, 177 (11.6 %) lines (i.e., CAPIII-S and CAPIV-S) exhibited a moderate to high level of adult plant resistance, defined here as a severity of 22.1 %, which is the mean plus one standard deviation of the severity exhibited by the resistant control Q21861. In 2010, 250 (16.5 %) lines (i.e., CAPI-S and CAPII-S) exhibited a moderate to high level of adult plant resistance, defined in this year as a severity of 0.7 % (Fig. 1b). Of these, 33 (2 %) and 31 (2 %) lines in 2009 (i.e., CAPIII-S and CAPIV-S) and 2010 (i.e., CAPI-S and CAPII-S) were found highly resistant with their severity equal to or lower than the mean resistant control of 13.7 and 0.2 %, respectively. The correlation coefficient (*r*) between the seedling disease score and adult plant severity was positive, but very low ranging from 0.06 for CAPI-S to 0.22 for CAPIII-S.

### AM

**Fig. 2:**
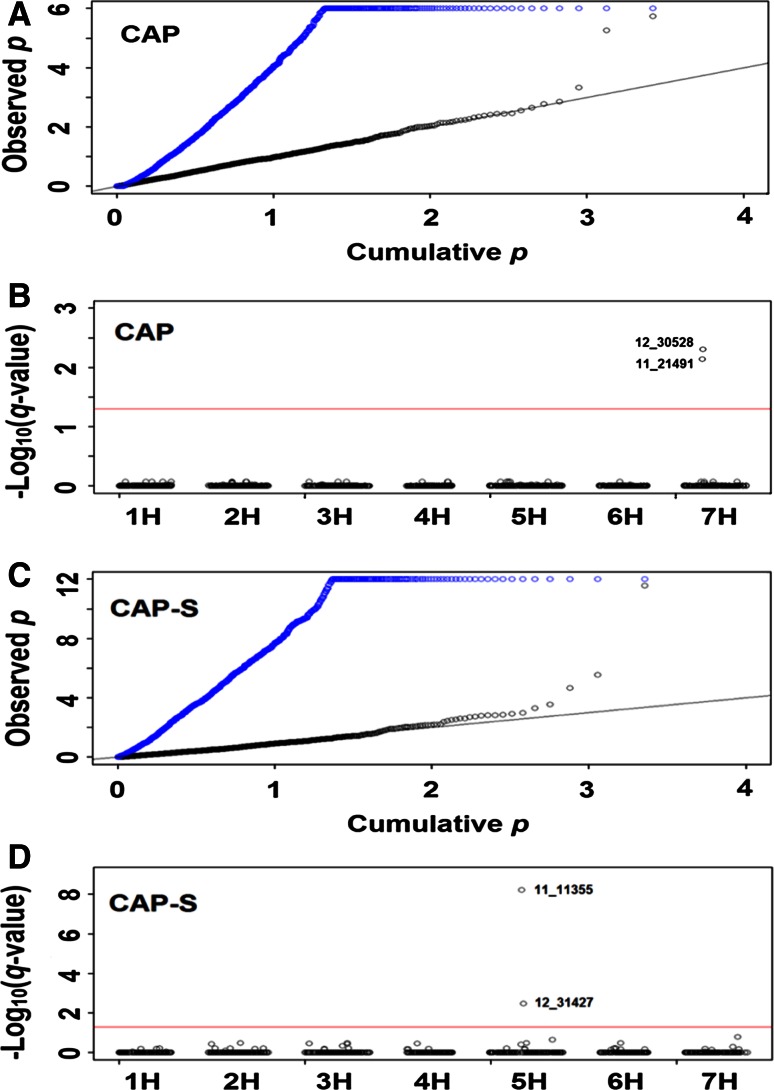
Genome-wide association of seedling and adult plant resistance to stem rust race TTKSK in US barley breeding germplasm. Quartile-quartile plots of naive (*in blue*) and corrected (*in black*) association *p* values for **a** seedling resistance in the complete panel (CAP) and **c** adult plant resistance in the complete spring panel (CAP-S). Genome-wide AM of **b** seedling resistance in CAP and **d** adult plant resistance in CAP-S. *Red horizontal line* indicates the threshold false discovery rate (FDR) = 0.05. The markers significantly associated with the resistance are labeled

Quantile–quantile (QQ) plots of cumulative (expected) and observed *p* values were used to show the effective control of population structure with the MLM. QQ plots (Fig. 2a, c) revealed that *p* values from the naive model were highly skewed from the straight line *y* = *x*, indicating the presence of extensive population structure within each panel. In stark contrast, plots of *p* values from the MLM mostly followed the straight line *y* = *x*, indicating that the correction of substructure in each panel was achieved (Fig. 2a, c).

AM was conducted in the complete (CAP) and individual yearly (CAPI–CAPIV) panels for seedling resistance and in the complete spring (CAP-S) and individual yearly spring (CAPI-S to CAPIV-S) panels for adult plant resistance. At the seedling stage, two neighboring SNP markers (0.8 cM apart) on chromosome 7H (11_21491 and 12_30528) were found significantly associated with resistance in CAP; however, neither of them was found associated in any of the individual yearly panels (Table 2; Fig. 2b). The high LD (*D*′ = 0.99) found between SNP markers 11_21491 and 12_30528 indicate that they are likely detecting the same locus. The most significant one found was 12_30528 (Table 2; Fig. 2b); thus, the resistance QTL was named *Rpg*-*qtl*-*7H*-*12_30528.* The phenotypic variation explained by each of these SNP markers was very low at 0.006. *Rpg*-*qtl*-*7H*-*12_30528* maps about 40 cM proximal to the well-characterized stem rust resistance locus *Rpg1* on chromosome 7H based on the consensus map of Muñoz-Amatriaín et al. (2011).

**Table 2:** SNP markers found significantly associated with resistance to stem rust race TTKSK at the seedling and/or adult plant stages in different panels of US barley breeding germplasm

SNP marker	Chrom.	Position^a^	Seedling					Adult				
			CAPI	CAPII	CAPIII	CAPIV	CAP	CAPI-S	CAPII-S	CAPIII-S	CAPIV-S	CAP-S
11_11355	5H	86.6	–	–	–	–	–	2 × 10^−3b^(0.03)^c^	2 × 10^−3^(0.04)	2 × 10^−8^(0.04)	–	6 × 10^−9^(0.02)
12_31427	5H	90.8	–	–	–	–	–	–	7 × 10^−3^(0.02)	3 × 10^−2^(0.02)	–	3 × 10^−3^(0.01)
12_10930	5H	94.4	–	–	–	–	–	–	1 × 10^−2^(0.02)	–	–	–
12_11106	5H	94.4	–	–	–	–	–	3 × 10^−2^(0.02)	–	–	–	–
12_21497	5H	94.4	–	–	–	–	–	1 × 10^−2^(0.03)	–	–	–	–
11_21491	7H	48.9	–	–	–	–	7 × 10^−3^(0.006)	–	–	–	–	–
12_30528	7H	49.7	–	–	–	–	5 × 10^−3^(0.006)	–	–	–	–	–

^a^Genetic position (in cM) of marker on chromosome

^b^ *q* value (multiple testing corrected *p* value)

^c^Variation explained by the individual SNP marker

**Fig. 3:**
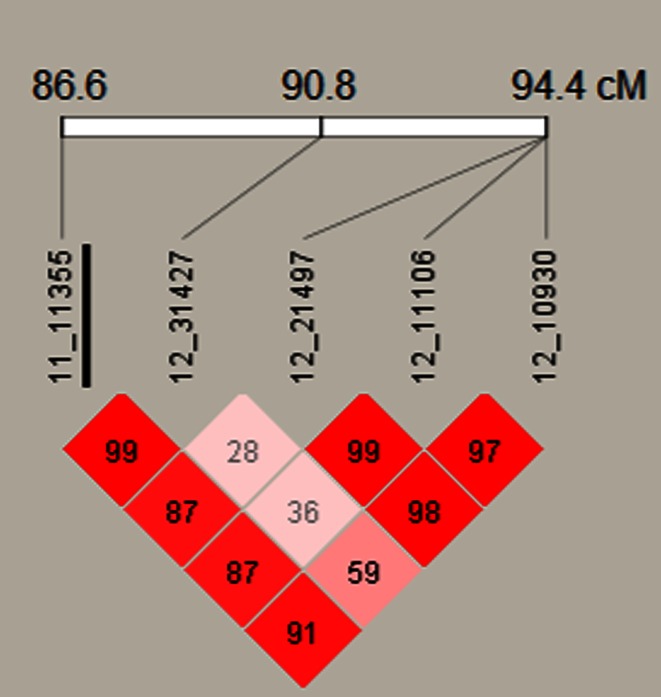
Heat map of LD among SNP markers significantly associated with adult plant resistance to stem rust race TTKSK on barley chromosome 5H. The number in each *square cell* is the pair-wise LD calculated in *D*′. The most significantly associated SNP marker (11_11355) is *underlined*

At the adult plant stage, two SNP markers on chromosome 5H (11_11355 and 12_31427) were found significantly associated with resistance in the complete spring panel CAP-S (Table 2; Fig. 2d). In the individual yearly spring panels, marker 11_11355 was significantly associated with resistance in CAPI-S, CAPII-S and CAPIII-S, whereas 12_31427 was detected as significant only in CAPII-S and CAPIII-S. In addition to these two associated markers, three other SNP markers (12_10930, 12_11106, and 12_21497) mapping to the same position on chromosome 5H were found significant, but only in one yearly panel (Table 2). Together, these five associated markers spanned a genetic distance of 7.8 cM. It is possible that the markers could be detecting the same QTL. To explore this possibility, LD (D′) among the five markers was examined. LD was high between the most significant SNP marker 11_11355 and the other four associated markers, ranging from 0.87 to 0.99 (Fig. 3). This resistance QTL was named *Rpg*-*qtl*-*5H*-*11_11355* for the most significant marker identified. All of these five associated SNP markers explained a small portion of the phenotypic variation, ranging from 0.01 to 0.04. *Rpg*-*qtl*-*5H*-*11_11355* maps at least 30 cM distal to the complex stem rust resistance locus r*pg4/Rpg5* on chromosome 5H based on the consensus map of Muñoz-Amatriaín et al. (2011).

### Allele effect of *Rpg*-*qtl*-*5H*-*11_11355*

To estimate the allelic effect of *Rpg*-*qtl*-*5H*-*11_11355*, the reduction of rust severity (in percent) for each program was calculated under high (2009) and low (2010) disease pressure (Table 3). Under high disease pressure, this QTL alone lowered disease severity by 3.7–21.1 % across eight spring breeding programs. Under low disease pressure, the QTL reduced disease severity 23.2–55.0 %. In general, the favorable (i.e., resistance) allele was fairly common across most breeding programs, ranging in frequency from 10 to 77 % (Table 3).

**Table 3:** Allele effect of the stem rust race TTKSK adult plant resistance-associated QTL (*Rpg*-*qtl*-*5H*-*11_*11355) in reducing disease severity across eight spring breeding programs of US barley breeding germplasm

High disease year 2009 (CAPIII-S and CAPIV-S)								
	MN^a^	N6	AB	UT	BA	N2	WA	MT
SNP 11_11355								
Susceptible allele	42.5^b^ (90 %)^c^	42.5 (55 %)	39.5 (50 %)	44.6 (39 %)	41.9 (50 %)	34.4 (47 %)	37.8 (46 %)	39.9 (25 %)
Resistant allele	34.8 (10 %)	37.0 (44 %)	31.9 (50 %)	35.2 (60 %)	35.3 (49 %)	28.9 (52 %)	36.5 (53 %)	31.7 (75 %)
Disease reduction (%)	18.0	13.0	19.2	21.1	15.8	16.0	3.7	20.6

Low disease year 2010 (CAPI-S and CAPII-S)								
	MN	N6	AB	UT	BA	N2	WA	MT
SNP 11_11355								
Susceptible allele	8.2 (85 %)	7.6 (61 %)	4.6 (25 %)	8.7 (17 %)	7.4 (44 %)	6.1 (33 %)	7.1 (71 %)	5.7 (48 %)
Resistant allele	6.0 (15 %)	4.2 (39 %)	3.6 (73 %)	5.1 (77 %)	4.2 (55 %)	2.8 (63 %)	5.2 (27 %)	3.0 (52 %)
Disease reduction (%)	26.7	44.9	23.2	41.6	42.5	55.0	26.7	47.7

^a^University of Minnesota (MN), North Dakota State University-six-rowed (N6), USDA-ARS Aberdeen (AB), Utah State University (UT), Busch Agricultural Resources Inc. (BA), North Dakota State University-two-rowed (N2), Washington State University (WA), and Montana State University (MT)

^b^Average disease severity for lines having a specific homozygous allele at SNP locus 11_11355

^c^Frequency of having a specific homozygous allele at SNP locus 11_11355

## Discussion

Race TTKSK is a serious threat to wheat and barley production worldwide because of its wide virulence. From the seedling evaluation of advanced US barley breeding germplasm, only 12 (0.3 %) lines exhibited resistance comparable to the resistant control. This frequency of resistance is lower than that found by Steffenson et al. (2013) (2 %) in a diverse collection of cultivated, landrace and wild *Hordeum* accessions. From the field evaluation of this germplasm in Kenya, again only 33 (2 %) lines from CAPIII-S and CAPIV-S and 31 (2 %) lines from CAPI-S and CAPII-S exhibited a high level of resistance comparable to the resistant control of Q21861. Of these 64 highly resistant lines, 10 were from breeding program AB; 3 from BA; 5 from MN; 8 from MT; 13 from N2; 3 from N6; 10 from UT and 12 from WA. Since these resistant lines are advanced in the breeding program (F_4_ generation or later), they will serve as useful parents in breeding for resistance to African stem rust races.

Genome-wide AM is an efficient approach for identifying genes controlling important agronomic traits. BCAP developed the germplasm and genomic resources for conducting robust AM studies of many important traits in barley, including resistance to stem rust race TTKSK. In this study, two closely linked SNP markers (11_21491 and 12_30528) on chromosome 7H were found associated with seedling resistance and five linked SNP markers (11_11355, 12_31427, 12_21497, 12_11106 and 12_10930) on chromosome 5H were found associated with adult plant resistance (Table 2). Since stem rust attacks barley after the heading stage, the adult plant resistance QTL *Rpg*-*qtl*-*5H*-*11_11355* is the highest value in breeding. To further confirm this QTL, 365 randomly selected lines from BCAP germplasm were phenotyped again in Kenya in 2011. Although disease severity was again low (as it was in 2010), the AM analysis still identified SNP markers 11_11355 and 12_31427 as being significantly associated with adult plant resistance (H. Zhou and B. Steffenson, unpublished). Thus, *Rpg*-*qtl*-*5H*-*11_11355* is robust because it was detected in all panels, except CAPIV-S, and under both low and high disease pressure. From QTL analysis of the biparental mapping population Q21861/SM89010, Moscou et al. (2011) found this same region of chromosome 5H associated with adult plant resistance in addition to the major effect *rpg4/Rpg5* locus (Steffenson et al. 2009). The results from this and our study indicate that this chromosome 5H region is very important for conferring adult plant resistance to stem rust race TTKSK in barley. Since 11_11355 was always the most significant marker identified in this region (Table 2) and all four other significantly associated markers were in high LD with 11_11355 (Fig. 3), it is likely that it lies closest to the causal variant. The synteny of barley with the *Brachypodium* (http://www.phytozome.net/) and rice (http://rice.plantbiology.msu.edu/) genomes was exploited through BLAST searches of the EST sequence from which the SNP 11_11355 was derived. The top BLAST hit was a glycogen operon protein glgX in the rice genome. Additionally, a plant–microbe interaction related gene (RING-H2 finger gene) (Zeng et al. 2006) also was found just two genes downstream from glgX. The RING-H2 finger gene forms the *ATL* gene family and may function as E3 ubiquitin ligases in plant–microbe interactions (Zeng et al. 2006). In Arabidopsis, constitutive expression of *ATL2* can up-regulate defense-related genes (Serrano and Guzmán 2004). Over-expression of the RING-H2 gene *StRFP1* reduced lesion growth rate when challenged by the late blight pathogen *Phytophthora infestans* in potato (Ni et al. 2010). Therefore, RING-H2 is a potential candidate gene for conferring adult plant resistance to stem rust race TTKSK. Additional fine mapping studies in the region will help to validate this hypothesis.

The *rpg4*/*Rpg5* complex is the only locus known to confer resistance to race TTKSK in barley. This resistance gene complex was first identified from Q21861 (Steffenson et al. 2009), an Australian breeding line selected from a CIMMYT/ICARDA (Centro Internacional de Mejoramiento de Maíz y Trigo or International Maize and Wheat Improvement Center/International Center for Agricultural Research in the Dry Areas) barley nursery in Mexico. Q21861 was used as a parent in several Midwest barley breeding programs (MN, N6, N2) in the early 1990s when a minor stem rust epidemic caused by race QCCJ occurred in North America (Steffenson and Smith 2006). However, it is likely that this gene complex is not present in the BCAP germplasm. This contention is based on the facts that we did not identify any lines exhibiting the characteristic low seedling infection type of 0; to race TTKSK in the greenhouse; the lack of any signal in the estimated *rpg4*/*Rpg5* mapping position (~116 cM) in the consensus map of Muñoz-Amatriaín et al. (2011); and pedigree analysis of breeding programs. *Rpg*-*qtl*-*5H*-*11_11355* maps to a clearly different position on chromosome 5H—86.6 in our study and 68-77 cM in the consensus map of Muñoz-Amatriaín et al. (2011) (M. Muñoz-Amatriaín, personal communication).

The coverage for genome-wide AM depends on two factors: LD extent in the mapping panel and SNP marker density (Rafalski 2002). In BCAP germplasm, LD decays over a distance of 20–30 cM (Hamblin et al. 2010). After removing the confounding structure effects with the MLM in this study, LD was reduced to about 6.3–7.5 cM based on the mean of significant pair-wise marker associations, an alternative method for measuring LD (Zhou et al. 2012). Considering that the genetic length of the barley genome is 1,099 cM (Close et al. 2009), one would only need about 174 markers (1,099/6.3 = 174) to cover the whole genome using a genome-wide AM approach if LD extent and marker distribution were even across the genome. Thus, the 2,099 SNPs used in this study should be sufficient for representing most polymorphisms in the genome. In fact, even with 1,536 markers, Cockram et al. (2010) was able to map 15 morphological traits in a collection of 500 barley cultivars from the United Kingdom and positioned the gene controlling anthocyanin pigmentation to a 140 kb interval. Nevertheless, one should still be cautious in extending the results from AM analyses due to the low frequency of functional alleles and possible presence of uncovered genetic regions. Indeed, in this study, a low frequency of stem rust resistance was found in BCAP germplasm at both the seedling and adult stages. This low allele frequency can greatly reduce the power to detect associations (Myles et al. 2009). Additionally, there are still ten gaps between 5 and 10 cM in length in the barley SNP map (Zhou et al. 2012). Further studies should be done to increase the mapping resolution of *Rpg*-*qtl*-*5H*-*11_11355* and explore whether any causal variants lie in these gaps. The recent development of the 9K SNP chip for barley and also exome capture strategies could be useful in closing these gaps in the map (Comadran et al. 2012; Mascher et al. 2013).

The prerequisites for detecting significant associations include several key factors aside from the frequency of functional alleles, including high quality genotype and phenotype data to ensure the data represent each individual’s true characteristics; a sufficiently high marker density to cover the whole genome; a large panel size to provide detection power; a strong association between a causal variant and linked marker; and a sufficiently large QTL effect to ensure detection of the marker (Jannink and Walsh 2002; Monks and Kaplan 2000; Risch and Merikangas 1996). In this study, AM was conducted in four individual panels and one complete panel for both the seedling and adult plant resistance assays. At the seedling stage, no SNP marker was found significantly associated with resistance in any of the individual four panels; however, two closely linked markers on chromosome 7H (11_21491 and 12_30528) were found associated in CAP. In this case, the consistency of phenotype data marker coverage and overall LD were almost the same. Thus, the only apparent factor for detecting these two SNP markers in the CAP panel was the larger population size (3,840 vs. 960 for an individual panel). Although the minimum sample size needed for detection of significant markers varies for different traits, (i.e., different QTL effects, LD extent in the target region and the mode of gene action: dominant, recessive, additive, etc.), one should recognize that sample size may be an important reason for failing to detect a significant association.

The associated markers identified in this study explained only a small portion of the phenotypic variation; however, *Rpg*-*qtl*-*5H*-*11_11355* showed a fairly large effect in reducing stem rust severity (Table 3) and may therefore be useful in breeding. The selection of this locus alone can reduce disease severity up to 55.0 % under low disease pressure and up to 20.6 % under heavy disease pressure (Table 3). Partial resistance allows the pathogen to infect the plant and ramify to some extent. As a result, the selection pressure exerted on the pathogen by partially resistant cultivars is much reduced compared to major gene resistance and can extend the life of the resistance. SNP marker 11_11355 will be valuable for marker-assisted selection of this resistance type to race TTKSK or as a component of a larger genomic selection effort in barley breeding.

## Electronic supplementary material

Below is the link to the electronic supplementary material.

Supplementary material 1 (DOCX 15 kb)
