# Supplementary material for: Association mapping of stem rust race TTKSK resistance in US barley breeding germplasm
Source: Theor Appl Genet. 2014 Apr 8;127(6):1293–304. doi: 10.1007/s00122-014-2297-8 (PMC4035542; doi:10.1007/s00122-014-2297-8)
Supplement: Supplementary file 1 — Supplementary material 1 (DOCX 15 kb) [file 122_2014_2297_MOESM1_ESM.docx]

| **Supplemental Table 1.** Breeding programs of the Barley Coordinated Agricultural Project contributing germplasm to the association mapping panel for stem rust resistance. | | | | | |
| --- | --- | --- | --- | --- | --- |
| **Breeding program** | **Location** | **Breeder** | **Growth habit** | **Row type** | **Primary use** |
| University of Minnesota (MN) | St. Paul, MN | Kevin Smith | Spring | Six | Malting |
| North Dakota State University (N6) | Fargo, ND | Richard Horsley | Spring | Six | Malting |
| USDA-ARS Aberdeen (AB) | Aberdeen, ID | Don Obert | Spring | Six/two | Malting/feed |
| Utah State University (UT) | Logan, UT | David Hole | Spring | Six/two | Feed |
| Busch Agricultural Resources Inc. (BA) | Ft. Collins, CO | Blake Cooper | Spring | Six/two | Malting |
| North Dakota State University (N2) | Fargo, ND | Richard Horsley | Spring | Two | Malting |
| Washington State University (WA) | Pullman, WA | Steve Ullrich | Spring | Six/two | Malting/feed/food |
| Montana State University (MT) | Bozeman, MT | Tom Blake | Spring | Two | Malting/feed/food |
| Oregon State University (OR) | Corvallis, OR | Patrick Hayes | Winter/facultative | Six/two | Malting/feed/food |
| Virginia Polytechnic Inst. & State Univ. (VT) | Blacksburg, VA | Carl Griffey | Winter | Six | Feed |
